# Plasma rich in growth factors in alveolar ridge preservation: randomized, controlled clinical trial

**DOI:** 10.1007/s00784-026-06769-z

**Published:** 2026-03-23

**Authors:** Eduardo Anitua, Alia Murias-Freijo, Joseba Loroño, Markel Loroño, Antonio González-Mosquera, Lucia Anitua, Mohammad H. Alkhraisat

**Affiliations:** 1 Clinica Eduardo Anitua, Vitoria, Spain; 2 University Institute for Regenerative Medicine & Oral Implantology, UIRMI (UPV/EHU-Eduardo Anitua Foundation), Vitoria, Spain; 3 BTI Biotechnology Institute, Vitoria, Spain; 4 Clinica Dental Murias, Bilbao, Spain; 5 Biomedical Research, Department of Cell Biology and Histology, Medicine and Nursing School, University of the Basque Country UPV/EHU, Leioa, Spain; 6 Clinica Dental Loroño, Bilbao, Spain; 7 Clínica Antonio González Mosquera, A Coruña, Spain; 8 Oral and Maxillofacial Surgery, Oral Medicine and Periodontics Department, Faculty of Dentistry, University of Jordan, Amman, 11942 Jordan

**Keywords:** Alveolar ridge preservation, Bone regeneration, Tooth extraction, Platelet rich plasma, PRGF, Fibrin, Biologics

## Abstract

**Purpose:**

The objective of this study is to evaluate the efficacy of Plasma rich in growth factors (PRGF) compared to spontaneous healing in alveolar ridge preservation of the aesthetic zone.

**Methods:**

This randomized and controlled clinical trial included 46 patients requiring simple-tooth extraction in the aesthetic zone and subsequent implant placement. The extraction sockets were treated either with PRGF or allowed to heal spontaneously. Postoperative healing was assessed by pain, Landry's soft tissue healing index, and inflammation index at 3, 7, and 15 days after tooth extraction. Histomorphometric bone regeneration was evaluated in biopsies harvested after 12 weeks of healing. Dimensional alveolar ridge measurements were performed at baseline and 12 weeks. Any postoperative complications and adverse effects were registered. Statistical analysis was performed to assess the differences between the study groups.

**Results:**

PRGF significantly enhanced postoperative healing, as evidenced by statistically significant reductions in pain on day 3 (*p* = 0.036) and improved soft tissue healing on days 3 (*p* = 0.047), 5 (*p* = 0.012), and 7 (*p* = 0.027). No significant differences were observed between groups regarding postoperative inflammation. The new bone formation was significantly improved with PRGF treatment (*p* = 0.024), with a median of 36.1% (range: 15.8% to 58.9%) in the control group compared to 48.7% (range: 31.9% to 92.3%) in the PRGF group. PRGF contributed to improved dimensional stability of the alveolar ridge at 12 weeks.

**Conclusion:**

PRGF improved healing following tooth extraction by promoting soft tissue healing and reducing postoperative pain. Additionally, it enhanced bone regeneration and contributed to better preservation of the alveolar ridge.

**Supplementary Information:**

The online version contains supplementary material available at 10.1007/s00784-026-06769-z.

## Background

Blood plays a vital role in maintaining homeostasis across tissues and ensuring the proper function of organs [1, 2]. It is endowed with multifunctional mechanisms that support homeostasis and tissue integrity [2]. Blood can respond rapidly to life-threatening events such as infection and bleeding, through highly specialized sensing and action mechanisms [3]. The multifunctionality of blood components supports this act-fast mechanism [4, 5]. For example, recent research has shown that coagulation factors VII, IX and X have antibacterial properties against drug-resistant gram-negative bacteria [6].

Notably, blood clot formation is essential for tissue repair; any factor that accelerates clot loss—such as in the case of a dry socket—can lead to healing failure [5]. It provides a unique immunomodulatory environment conducive to tissue healing. Growth factors, cytokines and extracellular vesicles are one of those mechanisms tailored to guide cellular response and communication throughout the four phases of tissue healing: hemostasis, inflammation, proliferation and remodeling [5, 7]. Ultimately, this cascade of events leads to tissue repair and reflects the blood’s intrinsic ability to maintain stability and promote survival.

Given these regenerative capabilities, it is unsurprising that both researchers and clinicians are increasingly interested in harnessing blood healing properties for the treatment and management of various injuries [8]. A prominent example is the growing use of platelet-rich formulations in the management of extraction socket [9]. In fact, the first clinical trial evaluating leukocyte-free platelet-rich plasma (PRP) for alveolar socket preservation was published as early as 1999 [10].

Tooth extraction inevitably leads to a reduction of the alveolar ridge. Classic studies report an average horizontal reduction ranging from 2.5 to 4.5 mm and a vertical reduction of 1.5 to 2 mm, with the latter being more pronounced on the buccal aspect[11–13].. When extrapolated to the anterior aesthetic zone, these data make the use of alveolar ridge preservation (ARP) techniques necessary to overcome the consequences of alveolar ridge remodeling [14].

Numerous materials have been investigated for alveolar ridge preservation, including autografts [15, 16], xenografts [17–20], allografts [21–24], synthetic grafts [19, 25], and biologics [9, 26–29]. Although all these materials and techniques show favorable outcomes in terms of alveolar ridge preservation (ARP), there is no clear evidence on the ideal material for managing the post-extraction socket[9, 30, 31].

The management of hopeless teeth in the aesthetic zone should be integrated into a comprehensive treatment plan aimed at ensuring long-term stability and maximizing patient satisfaction [32]. Aesthetic outcomes are a critical determinant of patient satisfaction, especially in implant dentistry, where aesthetic risk analysis plays a central role in treatment planning [33]. This analysis classifies patients into low, moderate, and high-risk categories based on various factors, including the anatomy of the soft tissue and the alveolar bone crest. Among the distinguishing features, horizontal bone deficiency characterizes moderate-risk patients, whereas vertical bone deficiency is typically observed in high-risk patients [32]. Therefore, implementing interventions immediately after tooth extraction to prevent or minimize dimensional changes in the extraction socket and promote bone regeneration aligns with the goal of reducing aesthetic risk. These interventions are collectively known as alveolar ridge preservation (ARP) techniques [14].

ARP encompasses procedures such as guided bone regeneration, socket fillers, and socket sealing [31, 34, 35]. While several ARP strategies exist, no single intervention has been definitively proven superior. For instance, Macbeth et al. found no conclusive evidence favoring any specific ARP technique, and similarly, a meta-analysis by Avila-Ortiz et al. was unable to identify a superior ARP or socket seal approach [31, 34]. Nonetheless, the use of xenografts or allografts combined with an absorbable collagen membrane or sponge has been associated with the most favorable outcomes in terms of reducing horizontal dimensional changes [31]. When evaluating ARP procedures, four key domains could be considered: dimensional stability, bone regeneration, keratinized tissue formation, and postoperative complications [34]. In this context, autologous blood products (ABP) emerged as promising ARP interventions due to their positive effects on tissue healing and postoperative recovery [9].

ABP are autologous in origin, biocompatible and could be prepared in the dental clinic, providing formulations capable of stimulating tissue healing [9, 36]. In this context, PRGF has broad clinical applications in dentistry, orthopedics, dermatology, ophthalmology and assisted reproductive technology [10, 37–42]. The absence of leukocytes in the formulation modulates the inflammatory process, and the resulting fibrin matrix allows for the controlled release of growth factors such as PDGF, TGF-β, VEGF, IGF-1, and FGF [43]. These factors promote cell proliferation, osteoblastic differentiation, angiogenesis, and extracellular matrix synthesis—processes that are fundamental for the regeneration of the alveolar ridge following tooth extraction [43].

Thus, the preservation of bone volume and soft tissues following tooth extraction in the anterior aesthetic zone represents a clinical challenge [14]. The purpose of this randomized clinical trial has been the evaluation of the efficacy of PRGF as alveolar ridge preservation intervention in anterior aesthetic zone of the maxilla. The primary objective of the study is to determine the percentage of newly formed bone at 3 months post-extraction. Secondary objectives include assessing dimensional changes of the alveolar ridge in both horizontal and vertical directions at 3 months post-extraction, evaluating soft tissue closure during the healing process, and monitoring postoperative inflammation and pain.

## Methods

### Study design

The manuscript was prepared following the CONSORT guidelines for randomized clinical trials. The study was designed as a randomized, controlled, parallel-group clinical trial with the aim of evaluating the efficacy of plasma rich in growth factors (PRGF) in alveolar ridge preservation after simple tooth extraction in the anterior region of the maxilla. A total of 46 patients from five private dental centers in Spain were included. All participants gave their informed consent after being duly instructed about the objectives of the study, as well as the risks and benefits associated with it. All procedures performed in this study complied with the ethical principles of the “Declaration of Helsinki (Fortaleza, 2013)” and the clinical trial protocol was approved by the Basque ethical committee for research with medication (Study code: FIBEA-04-EC/19/ALV). Registry: clinicaltrial.gov, TRN: NCT04093583, Registration date: 16 September 2019. Patient recruitment was performed between January, 2020 and November, 2023.

### Study population

Subjects were included if they met the following criteria: Adult patients of both sexes requiring a simple tooth extraction in the aesthetic zone (from the upper left second premolar to the upper right second premolar) and the later placement of a dental implant in the same location. Patients had to have non-active periodontal disease, the buccal wall dehiscence had to be less than 25% and the alveolar socket depth had to be ≥ 7 mm. Patients were included if they were willing to be observed during the treatment period and sign the informed consent form.

Before patient’s enrollment, the eligibility criteria were modified to include the alveolar socket depth had to be ≥ 7 mm.

Participants were excluded from the study if they met any of the following conditions:

- Presence of an active infection.
- Loss of any bone wall in the extraction socket.
- Severe inflammation in the areas scheduled for extraction prior to surgery.
- Previous diagnosis of coagulopathy or autoimmune disease.
- Ongoing or prior treatment with radiotherapy, chemotherapy, immunosuppressive therapy, systemic corticosteroids, or anticoagulants within 30 days prior to inclusion.
- Regular use of NSAIDs or other anti-inflammatory medications.
- History of chronic hepatitis or liver cirrhosis.
- Positive markers for HCV, HBsAg, HIV-I/II, or Treponema pallidum (TP).
- Poorly controlled diabetes mellitus (glycated hemoglobin [HbA1c] > 9%).
- Patients undergoing dialysis.
- The presence of malignant tumors, hemangiomas, or angiomas in the extraction area.
- History of ischemic heart disease within the last year.
- Pregnancy or women of childbearing age not using effective contraception.
- Breastfeeding women.
- Presence of metabolic bone disease.
- Ongoing treatment with oral or intravenous bisphosphonates.
- Smoking more than 10 cigarettes per day.
- Any condition that would prevent participation in the study.

### Randomization, allocation concealment, and blinding

The random assignment of the two treatments was performed using block randomization in blocks of four. A list of random numbers was generated using a web-based tool (https://www.sealedenvelope.com/simple-randomiser/v1/list). Each patient enrolled at each center was assigned a consecutive identification number. Based on the randomization list, each treatment (PRGF or spontaneous socket healing) was linked to a specific patient number and placed in a sealed envelope, labeled only with the corresponding identification number. Randomization was performed by the responsible of monitoring the clinical trial. All study variables were analyzed by a blinded researcher who was different from the clinical evaluator. The histologist who performed the measurements was kept blind to the assigned treatment. To maintain blinding, all source documents were coded using only the patient number assigned for the study and the visit number. Similarly, the data collection notebook included only the patient number as identifying information and labeled treatments as either A or B. The correspondence between the treatment administered (PRGF^®^ or conventional) and the patient number was recorded in a separate document kept by the principal investigator at each center. For statistical analysis purposes, treatments were analyzed as A and B, with the actual correspondence between A/B and the specific treatment (conventional or experimental) revealed only after the statistical analysis was completed.

### Surgical intervention

After signing the informed consent form and confirming eligibility based on the inclusion and exclusion criteria, the tooth extraction procedure was scheduled.

Peripheral blood was collected from each patient via venipuncture using 9 mL sodium citrate tubes provided in the Endoret^®^ KMU 15 kits (BTI Biotechnology Institute, Vitoria, Spain). The collected tubes were centrifuged at room temperature at 580 g using the Endoret^®^ System V centrifuge (BTI Biotechnology Institute, Vitoria, Spain). The resulting plasma was transferred into sterile fractionation tubes following the manufacturer’s protocol.

The upper plasma fraction (F1), which contains a platelet concentration similar to that of peripheral blood, was used to prepare the autologous fibrin membrane. The lower plasma fraction (F2), comprising the 2 mL immediately above the buffy coat, was used to obtain the PRGF clot. Both F1 and F2 fractions were free of red blood cells and leukocytes. To activate both plasma fractions, 20 µL of PRGF activator (10% calcium chloride) was added per mL of plasma. The formation of the PRGF clot required approximately 8 min, while the autologous fibrin membrane required about 15 min. Both products were maintained at 37 °C in a heating oven (Plasmaterm, BTI Biotechnology Institute, Vitoria, Spain) until application, to promote coagulation cascade activation. Simultaneously, analytical screening was performed to detect transmissible infections. This included immunochromatographic testing for *Treponema pallidum* antibodies using a commercial test (Artron Laboratories Inc., Burnaby, Canada), as well as a combined test (Artron Laboratories Inc.) for the detection of hepatitis B surface antigen (HBsAg), hepatitis C virus antibodies (HCV), and HIV type I and II antibodies.

To perform the tooth extraction, the intervention area was first anesthetized by injecting 4% articaine hydrochloride with epinephrine 1:100,000 (Artinibsa, Laboratorios Inibsa, Barcelona, Spain). The extraction was carried out atraumatically using syndesmotomes, elevators, and forceps, with the goal of preserving the entire alveolar socket. Following tooth removal, the socket integrity was assessed using a CPUNC-15-millimeter periodontal probe (Hu-Friedy, Chicago, IL, USA).

At this moment, the randomization envelope was opened by the scrub nurse, and the assigned treatment was administered by the surgeon (test group: PRGF; control group: spontaneous healing). In the test group, both the fibrin membrane and the PRGF clot were applied. In the control group, the natural blood clot was left in place. In both groups, the respective materials were stabilized with simple sutures using non-absorbable 5/0 polyamide (Supramid, Serag-Wiessner GmbH & Co., Naila, Germany). No flap elevation or surgical edge approximation was performed in either group.

Following the surgical procedure, intraoral photographs were taken using a Canon EOS 90D digital SLR camera (Canon Inc., Tokyo, Japan). An impression of the upper jaw was obtained using fast-setting alginate (Hydrogum 5, Zhermack, Italy) and subsequently cast with type IV dental stone (Elite Rock, Zhermack, Italy). Additionally, a cone-beam computed tomography (CBCT) scan was performed using either the NewTom VGi EVO tomograph (NewTom, Verona, Italy) or the Carestream CS 8200 3D system (Carestream Dental LLC, Atlanta, GA, USA), depending on the equipment available at each dental center.

CBCT scans were standardized across centers, with a field of view (FOV) of 10 × 10 mm and a voxel size of 0.15 mm. Exposure parameters were set at 90 kV and 10 mA, with the exposure time adjusted according to the manufacturer’s standard recommendations.

Postoperative care instructions and recommendations were provided to all patients. Paracetamol 1 g every 8 h was prescribed for postoperative pain management. Metamizole 575 mg was permitted as rescue medication if additional pain control was required at a dose of 1 or 2 capsules every 8 h.

A resin Maryland bridge was fabricated in those cases that required the extraction of a central incisor, a lateral incisor or a canine. The bridge was not in contact with the gingival tissue.

### Follow-up visits

The trial consisted of seven scheduled visits: a diagnostic visit, a treatment visit, follow-up visits at 3-, 7-, and 15-days post-surgery, a 12-week visit, and a one-year follow-up visit. This manuscript reported the outcomes until the 12-week visit.

At the follow-up Visits 3-, 7-, and 15-days post-surgery, Postoperative pain and inflammation were assessed, the Landry soft tissue healing index was recorded, and any postoperative complications or adverse events were evaluated.

At 12-week visit, an alginate impression and CBCT scan were obtained, and intraoral photographs were taken. Soft tissue healing was evaluated using the Landry soft tissue healing index. Coinciding with the dental implant placement, a soft tissue biopsy was taken from the center of the alveolus using a 2mm diameter circular scalpel (Kai, Kai Industries Co., Japan). A bone biopsy was collected using a trephine drill with a 2 mm internal diameter (BTI Biotechnology Institute, Vitoria-Gasteiz, Spain). Subsequently, a BTI UnicCa^®^ implant was placed according to the manufacturer’s surgical protocol (BTI Biotechnology Institute, Vitoria-Gasteiz, Spain).

#### Outcome variables

The primary efficacy variable was defined as new bone formation, expressed as the percentage of vital bone. Secondary efficacy variables included: horizontal and vertical remodeling of the alveolar ridge, expressed in millimeters, assessed by superimposing DICOM files from CBCT scans and STL files from model scanning; Soft tissue healing, expressed on a 1 to 5 scale and evaluated using the Landry et al.[44] soft tissue healing index; Postoperative pain, expressed as a continuous variable and measured using a visual analog scale (VAS); Inflammation, assessed on a 0 to 3 scale through direct observation[45], and the height of the epithelial layer in µm and measured via biopsies. Safety variables included the occurrence or absence of surgical and postoperative complications and adverse effects.

The evaluation of pain was originally set to be measured on a daily basis during the first week after surgery. The protocol was modified to include that pain assessment was assessed during the scheduled follow-up visits (days 3, 7, and 15).

### Histological and histomorphometric analysis

Samples were immediately fixed in 4% buffered formalin and bone biopsies were processed for decalcification using an EDTA-based method [46]. Subsequently, the biopsies were dehydrated, embedded in paraffin, and sectioned into 5 μm slices. Bone sections were stained with May-Grünwald Giemsa, while gingival biopsies were stained using Hematoxylin and Eosin. Photographs were taken at 10× magnification, and histomorphometric analysis was performed using FIJI software [47]. The percentage of newly formed bone and epithelial thickness were assessed.

### DICOM and STL analyses

The dimensional changes of the extraction socket were measured using multimodal integration of DICOM files (visualizing the bone structure of the maxilla) and STL files (representing the contour of the alveolar ridge and teeth) within implant planning software (Implant Studio, 3Shape, Copenhagen, Denmark). Plaster models were digitized using the Trios 5 intraoral scanner (3Shape, Copenhagen, Denmark).

For measurements, the baseline STL file was used as a reference to perform horizontal and vertical assessments of the extraction socket at both baseline and 12-week CBCT scans. The baseline STL file was superimposed onto the DICOM files by registering common anatomical landmarks (teeth) in the implant planning software. Once the DICOM and STL files were merged, the images were reoriented in the axial, sagittal, and coronal planes to visualize the full length of the alveolus. In the axial view, a line was drawn through the center of the alveolus, generating a sagittal view in which the longitudinal axis of the tooth root was identified. From this axis, perpendicular lines were drawn at 1, 3, and 5 mm apical to the bone crest. Additionally, the distance from the buccal and palatal bone crests to their corresponding papillae was measured. To assess horizontal changes at 12 weeks, after superimposing the baseline STL file onto the 12-week CBCT, a line connecting the buccal and palatal papillae was drawn to determine the baseline level of the bone crest. The width of the vestibular ridge at 1, 3, and 5 mm apical to this level was then recorded. Vertical bone reduction was calculated by comparing the distances from the vestibular and palatal gingival margins to the bone crest, between the baseline and 12-week time points. These vertical measurements were taken at mid buccal and mid palatal with all image planes consistently oriented.

#### Patient-reported outcome measures (PROMs)

Postoperative pain was recorded on days 3, 7, and 15 after surgery using a 10 cm visual analog scale (VAS) provided to the patients, where 0 represented no pain and 10 indicated the worst imaginable pain.

#### Data analysis

##### Sample size calculation

The sample size was calculated to detect a 27.57% difference in post-extraction socket bone regeneration between the control group and the PRGF group, assuming a common standard deviation of 30%[28]. A confidence level of 95% (α = 0.05) and a statistical power of 80% (β = 0.20) were established. Assuming a 1:1 allocation ratio between the control and PRGF groups and an anticipated dropout rate of 15%, the final sample size was determined to be 46 subjects, with 23 in each group.

#### Statistical analysis

$$\:r=\frac{\left|\mathrm{Z}\right|}{\sqrt{\mathrm{n}}}$$

Quantitative data were expressed as means and standard deviations when normally distributed, and as medians with interquartile ranges when not. The normality of variable distributions was assessed using the Shapiro–Wilk test. Categorical data were expressed as absolute frequencies and percentages. Quantitative differences between groups were analyzed using the Student’s *t*-test or the Mann–Whitney *U* test, depending on whether the assumption of normality was met. Effect size for non-parametric test was described by calculating the correlation (r) value according to Pautz et al., [48] by the following equation:

$$\:d=\frac{M1-M2}{Sp}$$

Where *Z* is the z statistic given by the non-parametric test and *n* is the sample size.

Thresholds for *r* of 0.1, 0.3 and 0.5 indicated small, medium and large magnitudes respectively [49]. 

Effect size of parametric test was described by Cohen’s *d* value calculated by the following Eq. 4 [9].

$$\:V=\frac{\sqrt{{x}^{2}}}{n\left(k-1\right)}$$

where *d* is the Cohen’s *d*, *M1* and *M2* are the sample means for the two groups and *S*_*p*_ represents the pooled standard deviation.

Thresholds for Cohen’s *d* of 0.2, 0.5 and 0.8 indicated small, medium and large magnitudes respectively [49]. 

Categorical differences between groups were analyzed using the Chi-square test. For categorical variables, the Cramer’s V value was calculated by the following equation:

where $\:{\chi\:}^{2}$is the chi-square statistic, *n* is the total sample size, and *k* is the number of levels of the categorical variable.

Thresholds for *V* of 0.1, 0.3 and 0.5 indicated small, medium and large magnitudes respectively [49]. 

A *p*-value ≤ 0.05 was considered statistically significant. Statistical analysis was performed using IBM SPSS Statistics, version 29 (IBM Corp., Armonk, NY, USA).

## Results

**Fig. 1:**
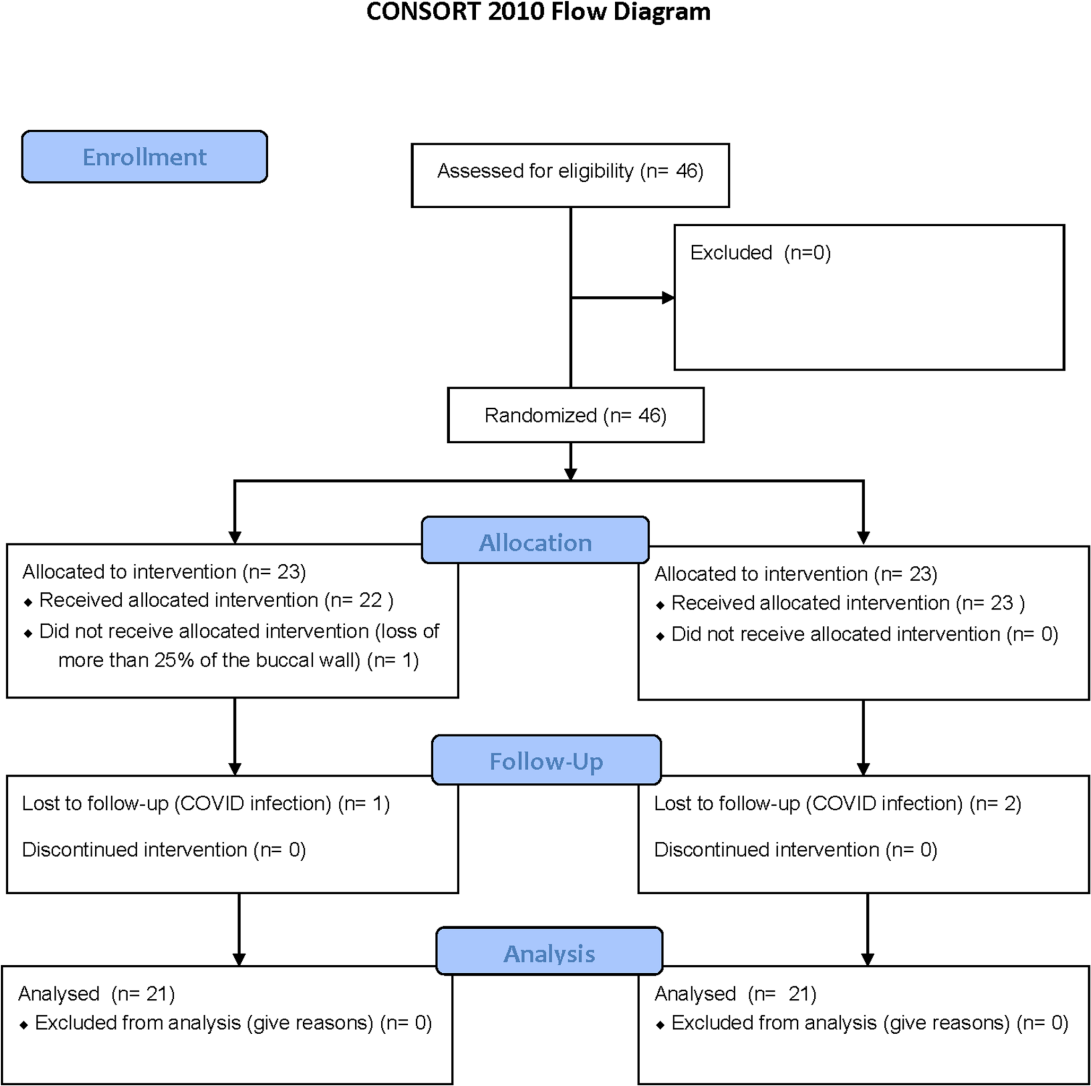
Study flow chart

Five private clinical centers in Spain participated in this clinical trial on a competitive recruitment basis. The distribution of patient recruitment was as follows: Center A enrolled 3 patients, Center B enrolled 30 patients, Center C enrolled 5 patients, Center D enrolled 3 patients, and Center E enrolled 5 patients. Figure 1 illustrates the flow chart of the clinical trial. A total of 46 patients were randomized into two groups: 23 patients in the control group and 23 in the test group. During the treatment visit, one patient in the test group did not receive the allocated intervention due to the loss of more than 25% of the buccal tissue and was subsequently withdrawn from the study. Additionally, three patients—two from the control group and one from the test group—were lost to follow-up due to COVID-19 infection. As a result, the final analysis included 21 patients in each group.

Table 1 summarizes the baseline characteristics of the study population. The study included 28 female patients—15 in the control group and 13 in the test group—and 17 male patients—8 in the control group and 9 in the test group. These sex differences were not statistically significant. The median age was 61 years in the control group and 55 years in the test group (*p* = 0.242). Five patients across both groups were smokers (< 10 cigarettes per day), with no significant difference between groups (*p* = 0.858). Alcohol consumption was reported by two patients—one in each group—and this difference was also not statistically significant (*p* = 0.977).

**Table 1:** Baseline characteristics of the study population

Variable		Control	Test	*p*-value
**Sex**	Female	15	13	0.677^a^
	Male	8	9	
**Age (years; median and range)**		61(21 to 74)	55 (30 to 78)	0.242^b^
**Smoking**	Yes	3	2	0.858^a^
	No	20	20	
**Alcohol consumption**	Yes	1	1	0.977^a^
	No	22	21	
**Reason to extract (unsavable tooth)**	Caries	5	2	0.220^a^
	Fracture	15	19	
	Periapical lesion	0	1	
	External root resorption	1	0	
	Defectous restoration	2	0	
**Complications at tooth extraction**	Tooth fracture	2	1	0.715^a^

a: Pearson test

b: Mann-Whitney test

**Fig. 2:**
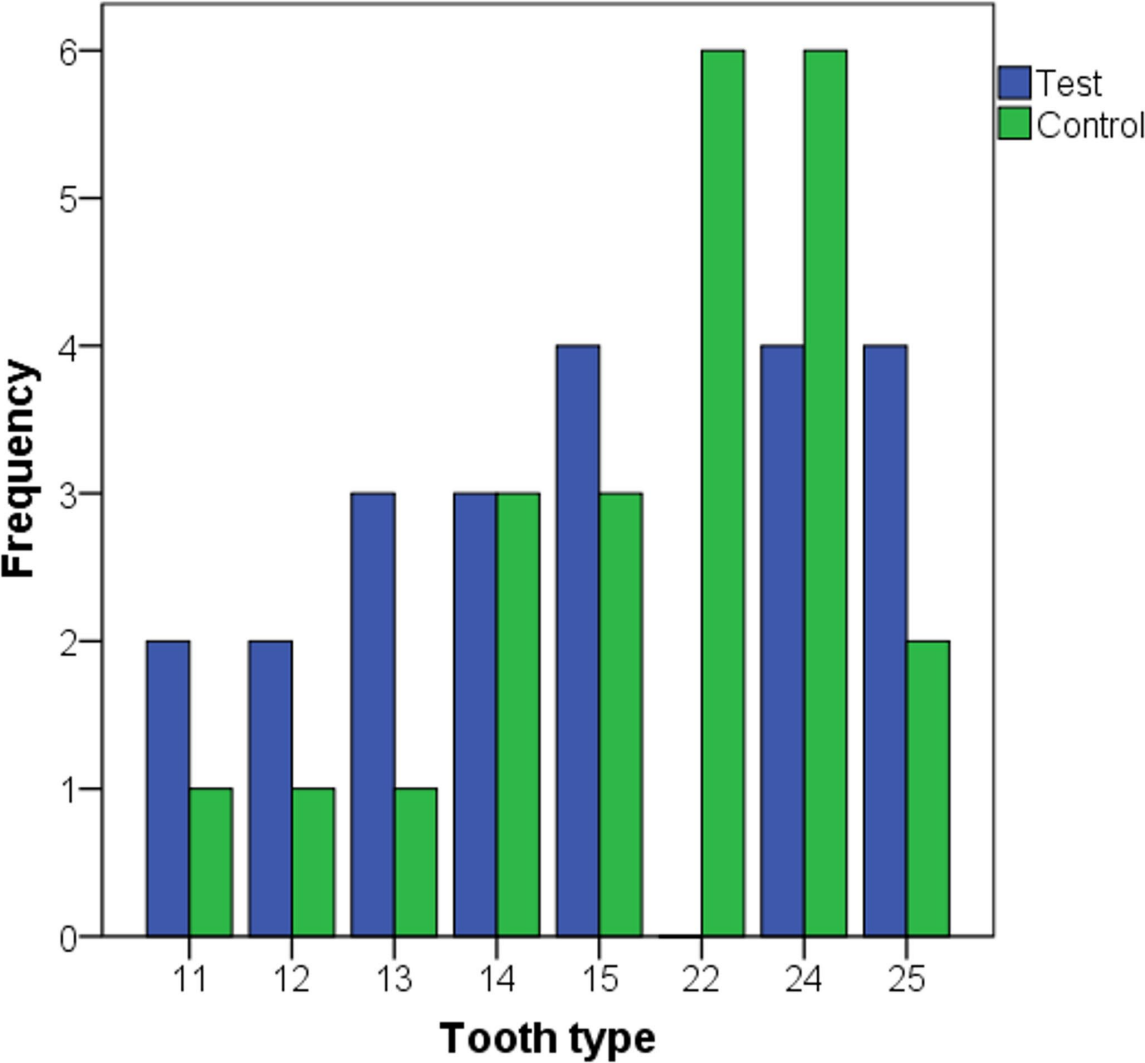
Location of extracted teeth according to the study groups. Teeth were numbered following the FDI tooth numbering system

Figure 2 presents the types of extracted teeth. There was no statistically significant difference between the study groups in this variable (Pearson test, *p* = 0.263). The reasons for tooth extraction are detailed in Table 1, with the most common indications being tooth fracture, followed by carious lesions. These distributions were not significantly different between the groups (*p* = 0.220). During the extraction procedures, tooth fractures occurred in three cases, two in the control group and one in the test group, with no statistically significant difference observed (*p* = 0.715).

### Post-operative healing

**Fig. 3:**
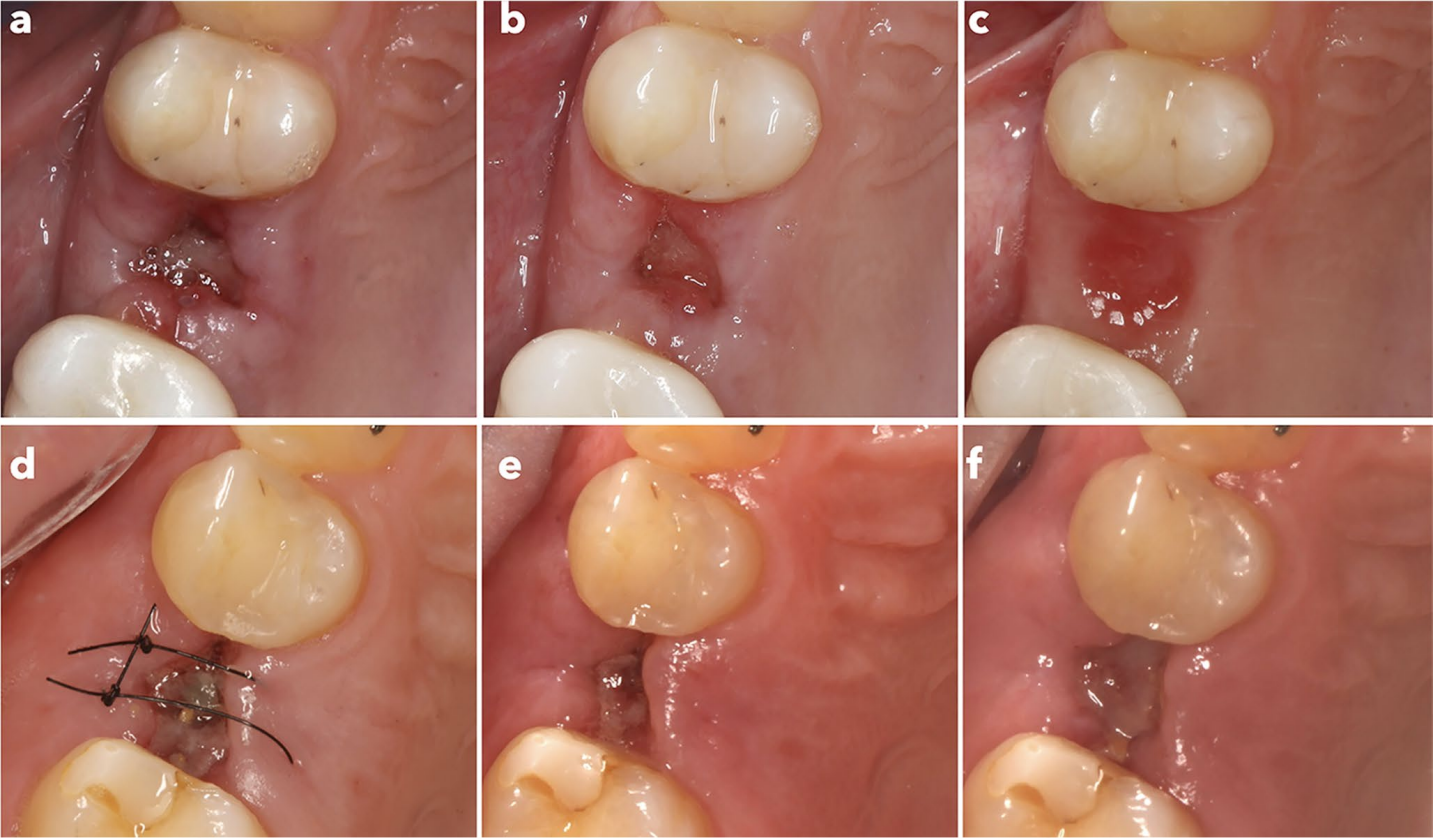
Soft tissue healing. Test group: (**a**) 3 days after surgery, (**b**) 7 days after surgery and (**c**) 15 days after surgery. Control group: (**d**) 3 days after surgery, (**e**) 7 days after surgery and (**f**) 15 days after surgery

Post-operative healing was evaluated using Landry’s soft tissue healing index, along with assessments of inflammation and pain scores (Table 2; Fig. 3). No infection events were observed in any of the patients.

#### On day 3 after surgery 

Data from two patients were missing due to non-attendance at the follow-up visit. Patients in the control group reported higher pain levels (median: 1, range: 0 to 7) compared to the test group (median: 0, range 0 to 2). This difference was statistically significant (*p* = 0.036). Effect size r had a value of 0.3 (95%CI: 0 to 0.6) and indicated a moderate effect. Regarding soft tissue healing, the Landry index showed that in the control group, 2 patients (10.5%) experienced poor healing, 11 (57.9%) had good healing, and 6 (31.6%) had very good healing. In contrast, none of the patients in the test group exhibited poor healing. Healing was assessed as good in 6 patients (28.6%), very good in 13 (61.9%), and excellent in 2 patients (9.5%). The difference in healing outcomes between the two groups was statistically significant (*p* = 0.047). The adjusted Cramer’s V had a value of 0.4 (95% 0 to 0.7), indicating a medium size effect. Regarding inflammation, none of the patients in the test group exhibited signs of inflammation. In the control group, two patients presented with slight inflammation, characterized by mild swelling and firmness without blurring of the facial planes. However, the difference between the groups was not statistically significant (*p* = 0.127).

#### On day 7 after surgery

Pain scores did not differ significantly between the study groups (*p* = 0.973), with a median pain score of 0 reported in both the control and test groups. However, the soft tissue healing index revealed statistically significant differences between the groups (*p* = 0.012). The adjusted Cramer’s V had a value of 0.4 (95% 0 to 0.7), indicating a medium size effect. In the control group, healing was assessed as poor in 1 patient (4.8%), good in 4 patients (19.0%), very good in 13 patients (61.9%), and excellent in 3 patients (14.3%). In contrast, the test group demonstrated faster healing progression, with 3 patients (14.3%) showing good healing, 5 (23.8%) very good healing, and 13 (61.9%) excellent healing. Inflammation was absent in all patients across both groups.

#### On day 15 after surgery

Data from two patients, 1 in each group, were missing due to non-attendance at the follow-up visit. None of the patients in either group reported pain at this stage. Soft tissue healing continued to progress in both groups, with significantly faster healing observed in the test group (*p* = 0.027). The adjusted Cramer’s V had a value of 0.4 (95% 0 to 0.7), indicating a medium size effect. In the control group, healing was rated as good in 2 patients (10%), very good in 10 patients (50%), and excellent in 8 patients (40%). In the test group, all patients demonstrated either very good (4 patients, 20%) or excellent (16 patients, 80%) healing. Inflammation remained absent in all patients across both groups.

**Table 2:** Postoperative pain, inflammation and laundry’s soft tissue healing index

Time	Index type	Score	Control	Test	*p*-value
**Day 3**	Soft tissue healing index	2	2	0	0.047^a^
		3	11	6	
		4	6	13	
		5	0	2	
	Inflammation	0	17	21	0.127^a^
		1	2	0	
	Pain (median and range)		1 (0 to 7)	0 (0 to 2)	0.036^b^
**Day 7**	Soft tissue healing index	2	1	0	0.012^a^
		3	4	3	
		4	13	5	
		5	3	13	
	Inflammation	0	21	21	-
	Pain (median and range)		0 (0 to 4)	0 (0 to 1)	0.973^b^
**Day 15**	Soft tissue healing index	3	2	0	0.027^a^
		4	10	4	
		5	8	16	
	Inflammation	0	20	20	-
	Pain (median and range)		0 (0)	0 (0)	1.000^b^

a: Pearson test

b: Mann-Whitney test

### New bone formation and epithelial thickness

**Fig. 4:**
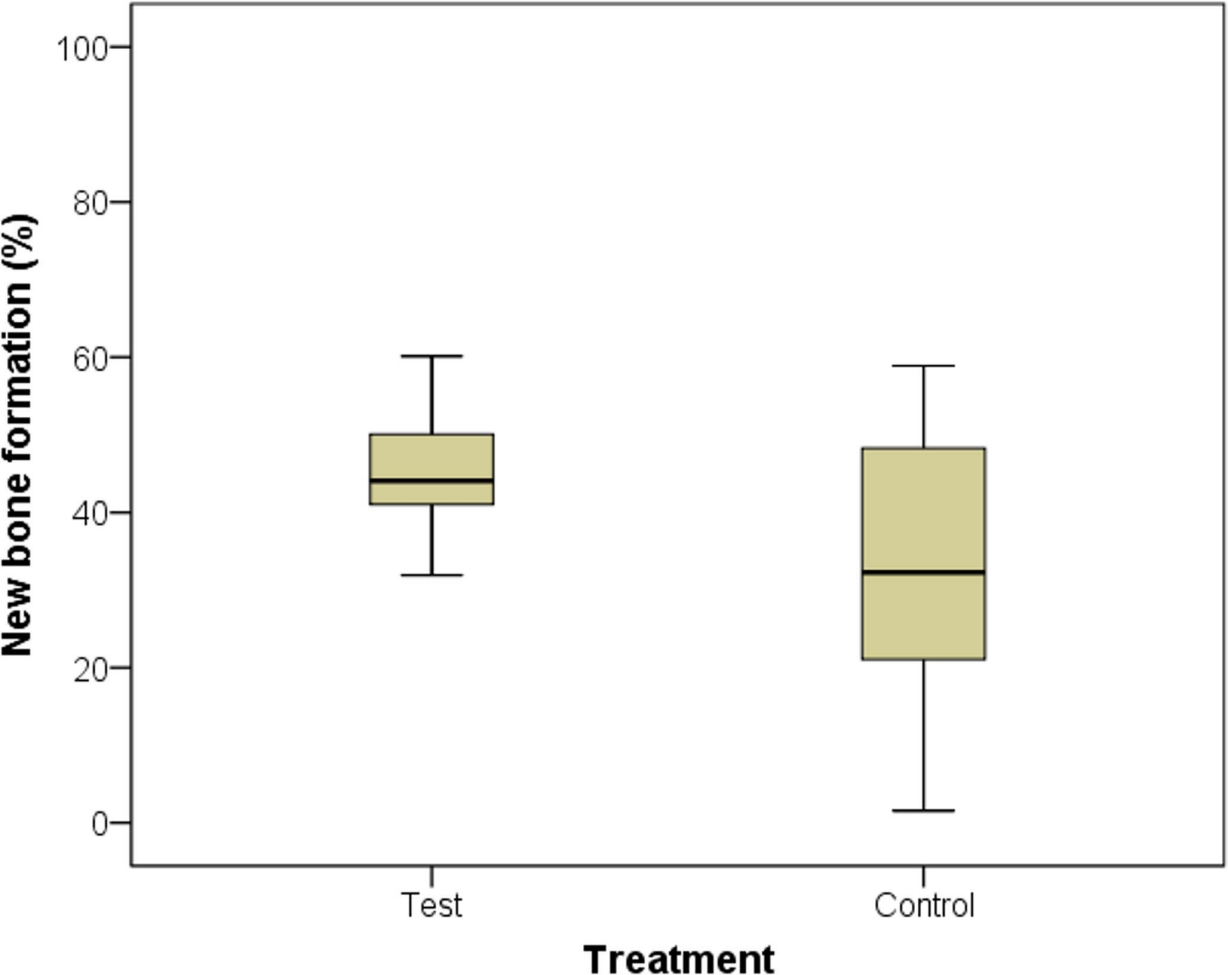
New bone formation after 12 weeks of healing in the test and control groups

**Fig. 5:**
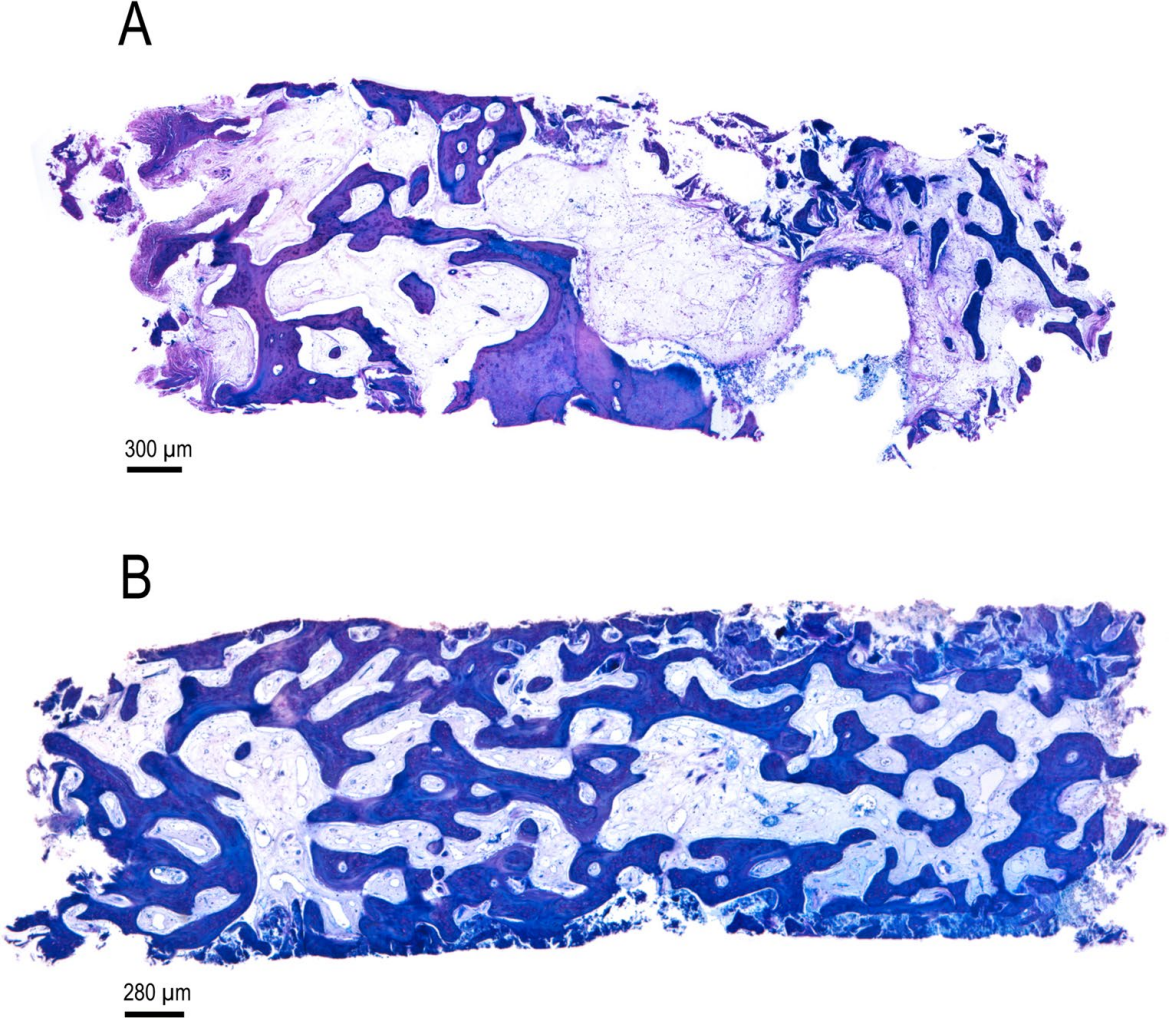
Histological images showing a bone biopsy harvested after 12 weeks in the control (**A**) and test (**B**) groups

The main variable in this study was new bone formation at 12 weeks (Fig. 4). Biopsies were obtained from 40 patients; however, one biopsy was not collected in the control group because bone drilling for dental implant placement was initiated prior to biopsy harvesting. Histological analysis revealed that mineralized bone tissue occupied 32.6% (range: 1.6% to 58.9%) of the sample area in the control group (Figs. 4 and 5). In contrast, the test group showed 48.7% (range: 31.9% to 92.3%) mineralized bone tissue. Statistical analysis using the Mann–Whitney test indicated a statistically significant difference between the groups (*p* = 0.008). Effect size r had a value of 0.4 (95%CI: 0.1 to 0.7) and indicated a moderate effect.

**Fig. 6:**
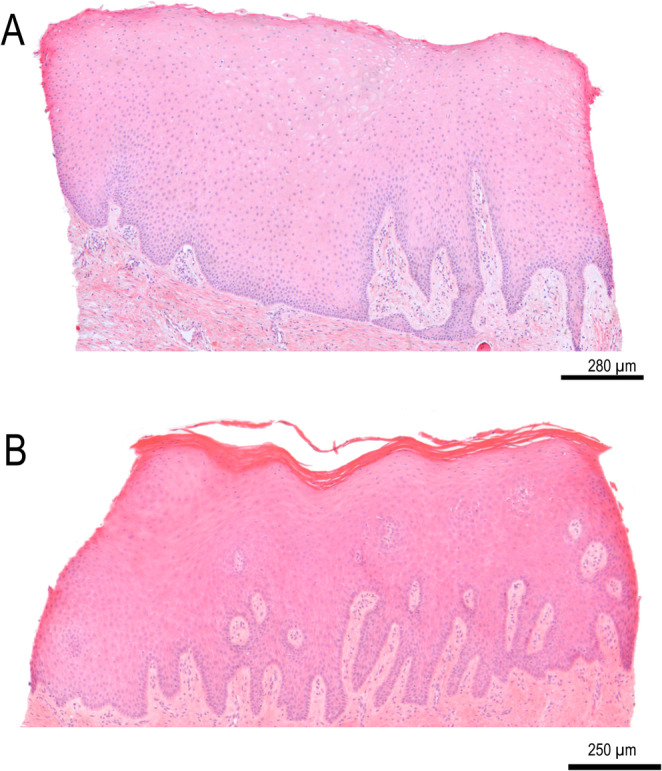
Histological images showing a gingival biopsy harvested after 12 weeks in the control (**A**) and test (**B**) groups

The soft tissue biopsies showed a similar epithelial thickness in both groups (Student t-test, p-value = 0.113) (Fig. 6). This thickness was 2030.1 ± 833.9 μm and 2450.7 ± 719.5 μm in the test and control groups, respectively.

### Dimensional changes in the alveolar ridge at the extraction site

Data from two patients, 1 in each group, were missing.

**Fig. 7:**
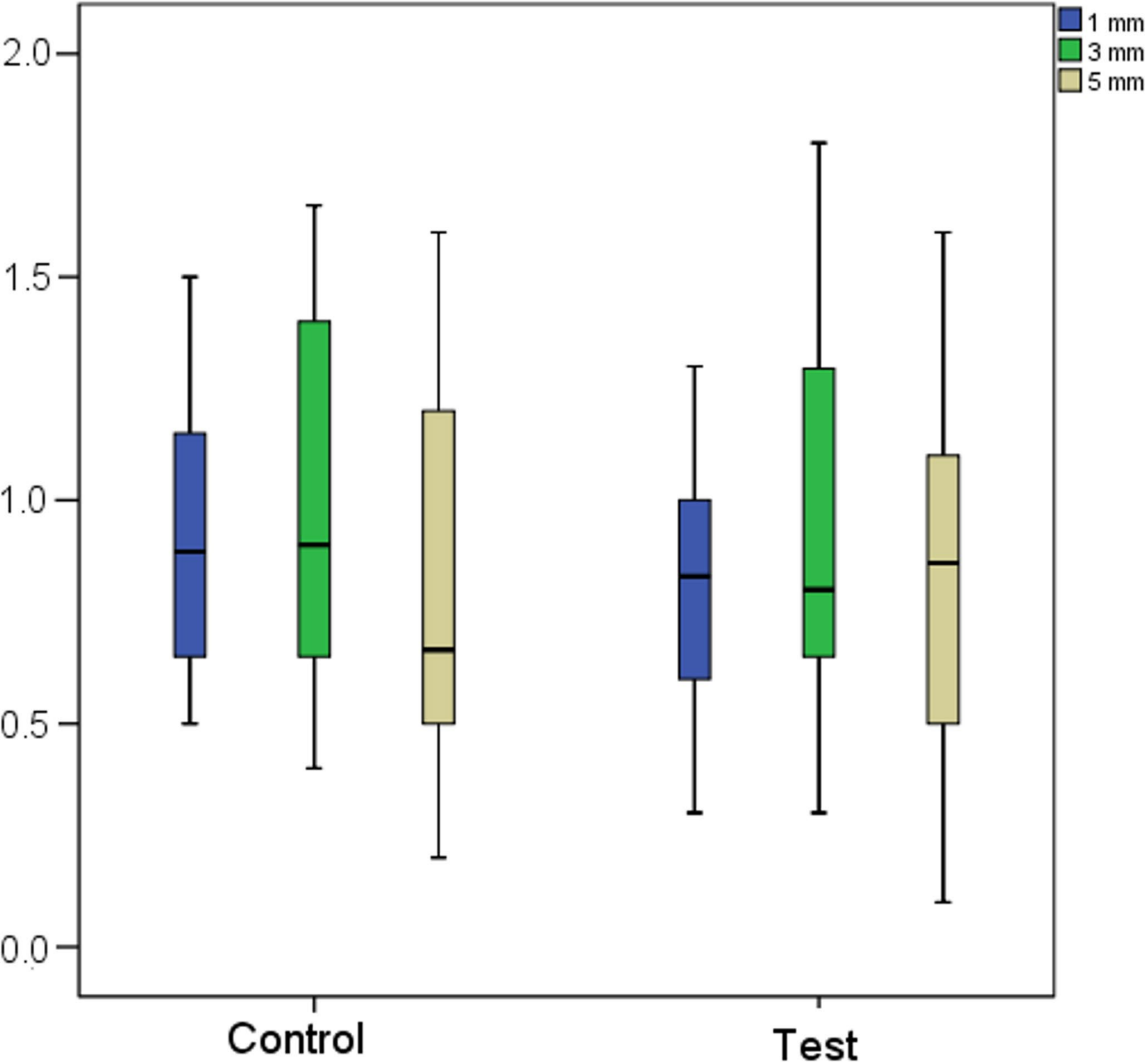
Buccal wall thickness at 1, 3 and 5 mm from the alveolar bone crest in the study groups

Figure 7 illustrates the buccal bone thickness measured at 1 mm, 3 mm, and 5 mm apical to the alveolar crest at baseline. Statistical analysis using the Mann–Whitney test revealed no significant differences between the control and test groups at any of the measured levels: 1 mm (*p* = 0.243), 3 mm (*p* = 0.587), and 5 mm (*p* = 0.914). Buccal wall thickness was also stratified according to the tooth type and allocated treatment (Online resource 1).

**Fig. 8:**
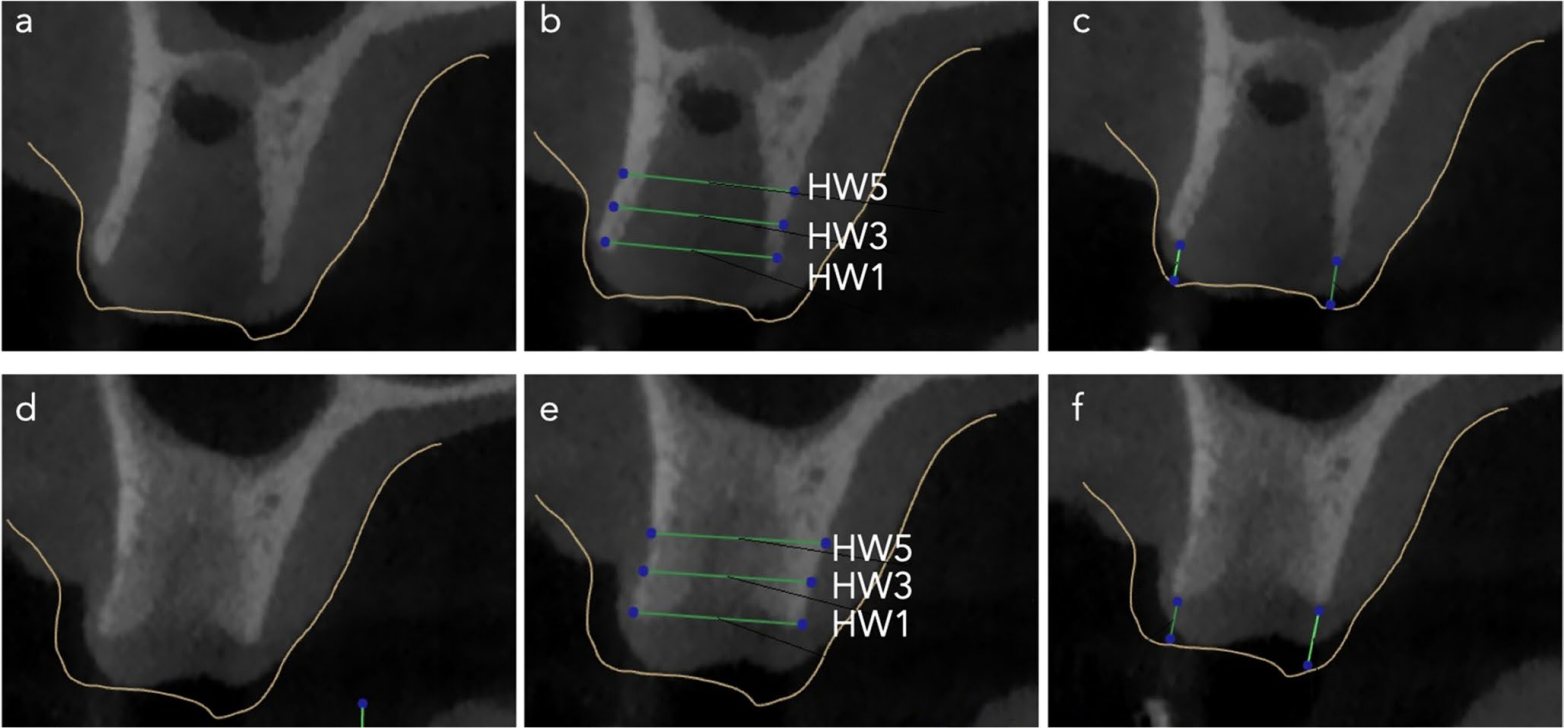
CBCT images of the extraction socket treated with PRGF for the evaluation of alveolar dimensional stability. (**a**) the superimposition of the STL file of the baseline cast model and the DICOM file of the baseline CBCT. (**b**) Baseline horizontal width measurements at 1 mm, (HW1), 3 mm (HW3) and 5 mm (HW5). (**c**) Baseline vertical measurement at midbuccal and mipalatal. (**d**) the superimposition of the STL file of the baseline cast model and the DICOM file of the 12-week CBCT. (**e**) 12-week horizontal width measurements at 1 mm, (HW1), 3 mm (HW3) and 5 mm (HW5). (**f**) 12-week vertical measurement at midbuccal and mipalatal

**Fig. 9:**
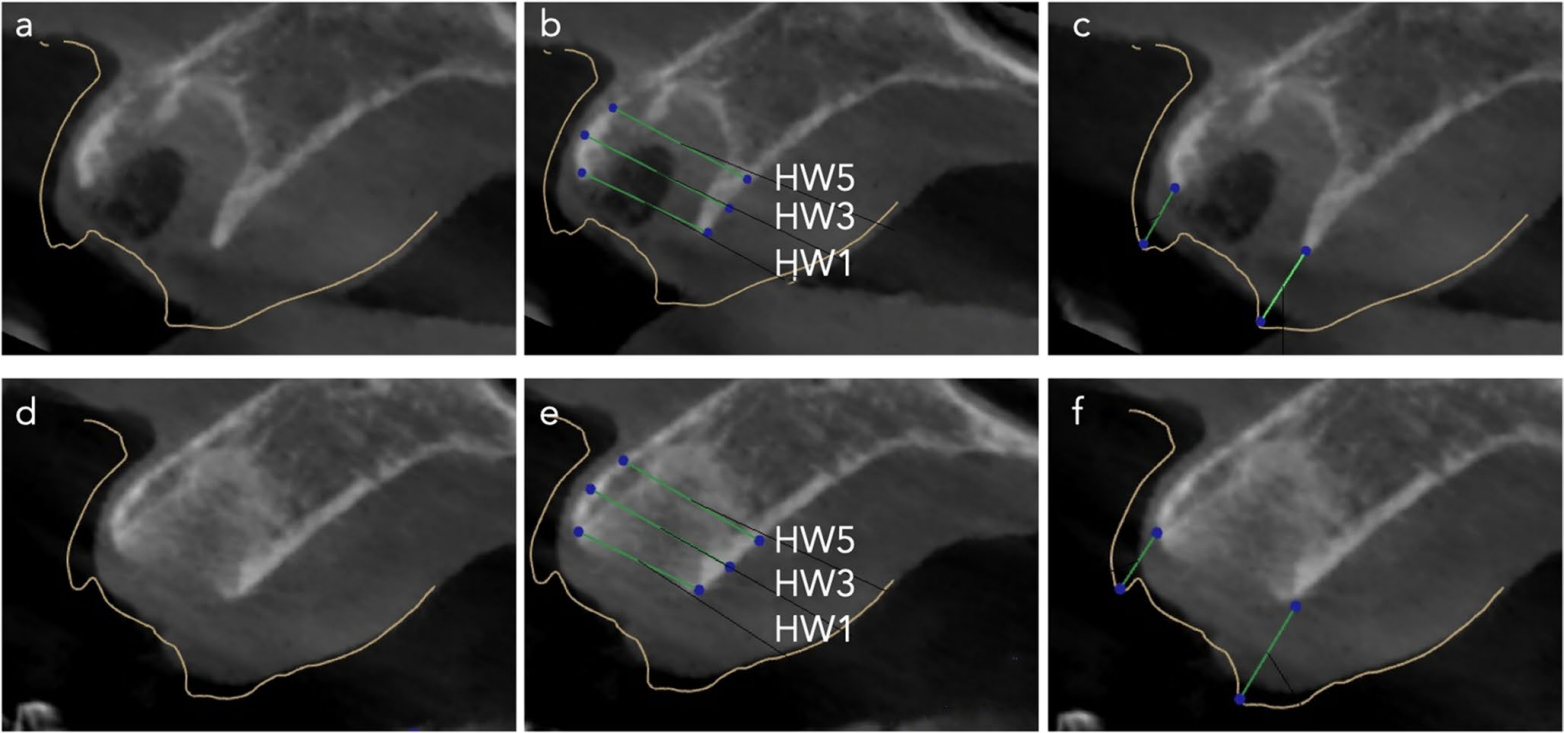
CBCT images of the extraction socket in the control group for the evaluation of alveolar dimensional stability. (**a**) the superimposition of the STL file of the baseline cast model and the DICOM file of the baseline CBCT. (**b**) Baseline horizontal width measurements at 1 mm, (HW1), 3 mm (HW3) and 5 mm (HW5). (**c**) Baseline vertical measurement at midbuccal and mipalatal. (**d**) the superimposition of the STL file of the baseline cast model and the DICOM file of the 12-week CBCT. (**e**) 12-week horizontal width measurements at 1 mm, (HW1), 3 mm (HW3) and 5 mm (HW5). (**f**) 12-week vertical measurement at midbuccal and mipalatal

The horizontal width (HW) of the alveolar ridge was measured at 1 mm (HW1), 3 mm (HW3), and 5 mm (HW5) apical to the alveolar crest (Figs. 8 and 9). Baseline measurements revealed no statistically significant differences between the study groups (Table 3). In the control group, HW1 decreased from a median of 8.9 mm (range: 6.3 mm to 14.0 mm)) at baseline to 7.1 mm (range: 1.3 mm to 9.2 mm)) after 12 weeks. In the test group, HW1 decreased from a median of 8.8 mm (range: 6.5 mm to 10.8 mm)) to 7.4 mm (range: 4.9 mm to 10.9 mm)) over the same period. Similarly, HW3 declined from a median of 9.4 mm (range: 6.7 mm to 14.8 mm) to 8.2 mm (range: 5.0 mm to 14.4 mm) in the control group and from 9.2 mm (range: 6.9 mm to 12.8 mm) to 8.0 mm (range: 6.1 mm to 12.2 mm) in the test group. At the HW5 level, the mean alveolar width decreased from 9.3 ± 1.9 mm to 8.3 ± 2.0 mm in the control group and from 9.3 ± 1.8 mm to 8.8 ± 1.9 mm in the test group. These findings indicate a net reduction in alveolar ridge width during the healing period, with significantly greater losses observed in the control group at HW1 and HW3. Effect size r had a value of 0.4 (95%CI: 0.1 to 0.7) and 0.3 (95%CI: 0 to 0.6), respectively. These values indicated a moderate effect. Specifically, the median reduction in HW1 was − 1.8 mm (range: −6.2 mm to −0.2 mm) in the control group compared to − 0.7 mm (range: −3.5 to 1.0) in the test group. At HW3, the loss was − 1.1 mm (range: −3.0 mm to 0.4 mm) in the control group versus − 0.6 mm (range: −3.1 mm to 0.9 mm) in the test group. Although the reduction in HW5 was also greater in the control group (mean and standard deviation: −1.0 ± 1.0 mm) than in the test group (mean and standard deviation: −0.4 ± 1.0 mm), this difference was not statistically significant.

To evaluate vertical remodeling, the distance between the alveolar crest and the gingival contour was measured on both the buccal and palatal aspects. The gingival contour reference was consistently taken from the baseline STL file. An increase in this distance indicated a loss in the vertical dimension of the alveolar crest. At baseline, the buccal alveolar crest was positioned at a median distance of 2.6 mm in the control group and 2.1 mm in the test group (*p* = 0.317). After 12 weeks, this distance increased to a mean of 3.9 mm in the control group and 2.9 mm in the test group (*p* = 0.020). The net change showed a significantly greater reduction in the vertical height of the buccal crest in the control group compared to the test group (*p* = 0.004), with a mean loss of 1.2 mm versus 0.5 mm, respectively. Cohen’s d had a value of −1.0 (95%CI: −1.6 to −0.3), indicating a large effect.

At baseline, the palatal alveolar crest was positioned at 3.6 mm and 3.3 mm from the reference line in the control and test groups, respectively (*p* = 0.447). After 12 weeks, this distance increased to 4.9 mm in the control group, while it remained unchanged at 3.3 mm in the test group (*p* < 0.001). The net change indicated a significantly greater loss in the vertical dimension of the palatal crest in the control group (1.4 mm), whereas the palatal crest remained stable in the test group (*p* < 0.001). Cohen’s d had a value of −1.6 (95%CI: −2.4 to −0.9), indicating a large effect.

**Table 3:** Dimensional changes in the alveolar ridge during healing

Variable	Baseline			12-week follow up			Change		
	Control	Test	*p*-value	Control	Test	*p*-value	Control	Test	*p*-value
HW-1 mm	8.9 (6.3 to 14.0)^b^	8.8 (6.5 to 10.8)^b^	0.903^d^	7.1 (1.3 to 9.2)^b^	7.4 (4.9 to 10.9)^b^	0.234^d^	−1.8 (−6.2 to −0.2)^b^	−0.7 (−3.5 to 1.0)^b^	0.019^d^
HW-3 mm	9.4 (6.7 to 14.8)^b^	9.2 (6.9 to 12.8)^b^	0.0.978^d^	8.2 (5.0 to 14.4)^b^	8.0 (6.1 to 12.2)^b^	0.598^d^	−1.1 (−3.0 to 0.4)^b^	−0.6 (−3.1 to 0.9)^b^	0.035^d^
HW-5 mm	9.3 ± 1.9 ^a^	9.3 ± 1.8 ^a^	0.974^c^	8.3 ± 2.0 ^a^	8.8 ± 1.9 ^a^	0.421^c^	−1.0 ± 1.0 ^a^	−0.4 ± 1.0 ^a^	0.089^c^
Vertical distance to B	2.6 (1.1 to 5.4)^b^	2.1 (1.0 to 4.7)^b^	0.317^d^	3.9 ± 1.3 ^a^	2.9 ± 1.1 ^a^	0.020^c^	1.2 ± 0.8 ^a^	0.5 ± 0.7 ^a^	0.004^c^
Vertical distance to P	3.6 ± 1.5 ^a^	3.3 ± 1.0 ^a^	0.447 ^c^	4.9 ± 1.3 ^a^	3.3 ± 0.9 ^a^	0.000^c^	1.4 ± 1.0 ^a^	0.0 ± 0.6 ^a^	0.000^c^

HW: Horizontal width

B: Buccal bone crest

P: Palatal bone crest

a: mean ± standard deviation

b: median and range

c: Student test

d: Mann-Whitney test

## Discussion

Plasma rich in growth factors (PRGF), a type of ABP, is prepared using a specific protocol that yields a leukocyte- and erythrocyte-free plasma with 2 to 3 times the platelet concentration of peripheral blood [10, 37, 50]. PRGF has demonstrated clear benefits in enhancing soft tissue healing, reducing postoperative pain and complications, improving patients’ quality of life and satisfaction, and offering advantages for medically compromised patients [51, 52]. This clinical trial has further investigated the use of autologous blood products (ABPs), particularly PRGF, as an intervention for alveolar ridge preservation. It evaluated bone regeneration, alveolar dimensional stability, soft tissue healing, and postoperative pain and inflammation.

Significantly greater new bone formation in the extraction socket was observed in the PRGF group compared to unassisted healing. These findings align with a meta-analysis reporting a positive effect of PRGF on new bone formation in extraction sockets, showing a standardized mean difference (SMD) of 1.44 (95% CI: 0.84 to 2.03) [53]. Additionally, they are consistent with the systematic review and meta-analysis by Caponio et al.[54], which reported that ABP has a statistically significant and positive effect with an SMD of 1.77 (95% CI: 1.47–2.06; *p* < 0.001). Several mechanisms may help explain how PRGF enhances tissue formation. All regenerative processes—regardless of tissue type—begin with the formation of a blood clot, which subsequently undergoes remodeling and transformation into an extracellular matrix that orchestrates new tissue formation and defect repair [55]. Unlike clots containing leukocytes and erythrocytes, PRGF has been shown to support the proliferation of fibroblasts and osteoblasts within its fibrin clot, thereby promoting its remodeling into a functional extracellular matrix[56, 57]..

Another interesting aspect of ABP is their dual role: they not only serve as a scaffold supporting cell adhesion and migration, but also release a supernatant rich in active biomolecules [43]. Recent research has shown that these two systems—the clot scaffold and its supernatant—exert distinct effects on bone progenitor cells [58, 59]. The diffusion of PRGF-derived supernatant into adjacent areas may increase the expression of the Runx2 gene, thereby promoting the proliferation of undifferentiated bone cells, while simultaneously inhibiting the expression of the SP7 gene, which is associated with osteoblastic differentiation. In contrast, direct contact between these cells and the PRGF fibrin clot enhances their differentiation into osteoblasts, as indicated by increased SP7 gene expression and elevated alkaline phosphatase activity [59]. Considering the documented chemotactic effect of PRGF on stem cells [60], the overall result of these biological mechanisms would be enhanced migration and concentration of stem cells at the bone defect site, thus supporting regeneration. Additionally, a recent proteomic study on human periodontal ligament stem cells (hPDLSCs) found that PRGF supernatant upregulates the expression of proteins involved in extracellular matrix organization, cell adhesion, proliferation, and migration. PRGF-derived fibrin activated several proteins that are linked to cellular activation, respiration, and electron transport. hPDLSCs cultured in contact with PRGF fibrin exhibited strong osteogenic potential, with differential protein expression related to ossification, tissue remodeling, morphogenesis, and cell migration [58]. Together, these findings suggest that PRGF-mediated mechanisms positively influence bone regeneration in post-extraction sockets. Torres et al. evaluated the influence of platelet-rich plasma on the healing dynamics of bone defects in rabbit calvaria during the first 8 weeks of healing [61]. Their study demonstrated that the application of platelet-rich plasma significantly accelerated new bone formation at the early healing stage (4 weeks) compared with spontaneous healing.

The complete biodegradability of PRGF, compared to conventional materials, may offer a clinical advantage by providing more space for new bone formation. For instance, Couso-Queiruga et al.[62] evaluated the use of deproteinized bovine bone mineral (DBBM) combined with collagen membranes (CM) as an alveolar ridge preservation (ARP) treatment in anterior teeth, grouping results by histomorphometric evaluation time. Reported new bone formation percentages were 13.53% (95% CI: 0–59.62%) at 3 months, 33.33% (95% CI: 0.49–56.21%) at 6 months, and 37.05% (95% CI: 9.53–68.85%) at 9 months. Similar findings have been reported in a randomized clinical trial (RCT) by Stumbras et al.[63] which assessed four treatment groups: DBBM + CM, FDBA + CM, PRGF, and spontaneous healing. At 3 months, histological biopsies showed that the use of PRGF alone in post-extraction sockets of the aesthetic zone resulted in 75.5% ± 16.3% mineralized bone. In comparison, sockets treated with DBBM + CM showed 20.3% ± 21.9%, those with FDBA + CM had 7.2% ± 8.6%, and the spontaneous healing group reached 46.4% ± 15.2%.

The dimensional loss of the alveolar ridge preservation after tooth extraction is a consequence of the resorption of the bundle bone and the alveolar walls [12]. Tooth extraction disrupts the blood supply and triggers osteoclastic activation and, consequently, bone resorption [64]. It has been estimated that the physiological remodeling process of the socket following tooth extraction has resulted in a weighted average loss of 3.87 mm horizontally and 1.67 mm vertically [14, 65, 66]. These figures represent a reduction of between 29% and 63% in the width of the alveolar ridge, and between 11% and 22% in its height [66]. As a result, the alveolar ridge tends to shift in apical and palatal directions [12, 14]. The new position of the buccal plate is the result of a dynamic balance between the anabolic process of bone formation and the catabolic process of resorption. Thus, the positive effect of PRGF on bone regeneration could explain its efficacy in minimizing the dimensional loss of the post-extraction socket. Specifically, by promoting de novo bone formation near the buccal plate, PRGF would help counteract the characteristic bone resorption process in this region[67].

Indeed, PRGF has significantly improved the dimensional stability of the alveolar ridge following tooth extraction. The control group exhibited greater horizontal width reduction at all measured points (HW1, HW3, HW5) compared to the test group, with statistically significant differences observed at HW1 and HW3, but not at HW5. These findings are consistent with those reported in the randomized controlled trial by Stumbras et al. [68], which showed horizontal ridge width reductions of − 1.61 ± 1.76 mm, − 0.83 ± 1.20 mm, and − 0.82 ± 0.86 mm at 1 mm, 3 mm, and 5 mm from the alveolar crest, respectively, following unassisted healing. In contrast, the PRGF-treated group demonstrated lower resorption values at the same levels, with reductions of − 1.25 ± 1.10 mm, − 0.22 ± 0.94 mm, and − 0.003 ± 1.18 mm, respectively. In this study, PRGF reduced alveolar ridge width loss by 1.1 mm. This effect is consistent with the findings reported in the Cochrane review by Atieh et al. [30], which meta-analyzed six studies involving 201 post-extraction sockets. That review compared the use of xenografts versus spontaneous healing and found a mean difference in alveolar ridge width loss of − 1.2 mm (95% CI: −1.8 to − 0.5; *p* = 0.0003) in favor of the xenograft group. However, a recent systematic review found no significant effect of using ABP on horizontal remodeling of the alveolar process compared to unassisted healing [69]. This observation underscores the need for well-designed randomized clinical trials.

Similarly, the net change revealed a significantly greater reduction in the vertical height of the buccal crest in the control group compared to the test group, with a mean loss of 1.2 mm versus 0.5 mm, respectively. A significantly greater vertical loss was also observed in the palatal crest of the control group (1.4 mm), whereas the palatal crest remained stable in the test group. Stumbras et al. also reported a statistically significant effect of PRGF in reducing vertical alveolar height loss, from − 0.86 ± 0.43 mm in the unassisted healing group to − 0.54 ± 0.86 mm in the PRGF group [68]. This is in line with a recent systematic review, which reported statistically significant differences favoring the use of ABP in terms of vertical remodeling of the alveolar process (mean difference = 0.57 mm [95% CI 0.22, 0.91]) [69]. In the Cochrane review, xenografts were associated with a mean difference in height loss of − 1.35 mm (95% CI: −2.00 to − 0.70; *p* < 0.0001), based on an analysis of six studies involving 184 participants and 201 extraction sites, when compared to unassisted healing [30].

This clinical trial reported improved and statistically significant soft tissue closure compared to spontaneous healing at 3, 7, and 15 days of evaluation. These findings are consistent with the results of a systematic review assessing the impact of plasma rich in growth factors in the healing of extraction socket [51]. Furthermore, Kings et al. have reported that PRGF accelerated soft tissue healing, decreased inflammation and halitosis in patients with alveolar osteitis compared to Alvogyl [70]. Similar effects have been also observed in clinical studies assessing PRGF in patients at risk of delayed wound healing [71–74].

Several studies have demonstrated the positive effect of PRGF on cell proliferation and migration (wound healing model), as well as on the synthesis of key proteins such as VEGF, HGF, TGF-β, and type I procollagen in fibroblasts [75, 76] Clinically, PRGF has been shown to be safe and effective in accelerating wound closure [77]. This beneficial effect is attributed to the absence of a pro-inflammatory signature in its composition, which promotes an anabolic—rather than catabolic—effect on fibroblasts, linked to the absence of leukocytes in its formulation [78]. Fibrin remodeling by fibroblasts, as well as extracellular matrix formation, is enhanced in PRGF clots, in contrast to the negative effects observed when leukocytes and erythrocytes are included [79]. PRGF is a biologically complex therapeutic, composed of multiple elements that contribute to favorable clinical outcomes in regeneration, such as the sustained release of growth factors including VEGF, PDGF, TGF-β1, and IGF-1; the presence of extracellular vesicles, mitochondria, and lipids [7, 75, 80]. All these components play a key role in modulating inflammation, promoting angiogenesis, and stimulating mesenchymal stem cells [81]. This more favorable immunological microenvironment may help attenuate the initial inflammatory response by promoting macrophage polarization from a pro-inflammatory (M1) phenotype toward an osteogenic (M2) phenotype, representing a significant advantage over the native blood clot [82]. As a result, early soft tissue closure and less pain have been observed when extraction socket was treated with the PRGF.

This randomized clinical trial presents several limitations that should be taken into account when interpreting its findings. Blinding was feasible for the histological analysis—the primary outcome—as well as for data analysis, but clinical assessors could not be blinded due to the visible color differences between treatment groups. Additionally, the COVID-19 pandemic impacted the progression of the trial, contributing to the withdrawal of three participants. Missing data was also caused by patient’s missing the scheduled visits to monitor the study variables.

Recruitment capacity varied across participating centers, influenced by differences in patient volume, which may have introduced variability in enrollment rates. Nevertheless, randomization was effective in controlling key confounding factors, such as buccal bone wall thickness, which showed no statistically significant differences between groups, thereby supporting the internal validity of the study.

However, the generalizability of these results to patients with advanced buccal wall loss or those with serious and/or uncontrolled systemic conditions remains uncertain. Further studies are warranted to confirm these outcomes in broader, real-world clinical settings. Further studies are needed to assess the impact of alveolar ridge preservation strategies on the clinical and esthetic outcomes of implant-supported rehabilitations.

## Conclusions

This clinical trial supports the efficacy of PRGF as a biological and autologous treatment for alveolar preservation following tooth extraction in the esthetic zone. The use of PRGF has demonstrated both clinical and histological efficacy, showing favorable and statistically significant results in terms of new bone formation, dimensional stability of the alveolar ridge, and soft tissue healing. Regarding patient-reported outcome measures (PROMs), the application of PRGF contributed to improved postoperative recovery, with lower pain levels. No surgical or postoperative complications, nor adverse effects, were reported in either group.

## Supplementary Information

Below is the link to the electronic supplementary material.

Supplementary Material 1

## Data Availability

The datasets used and/or analyzed during the current study are available from the corresponding author on reasonable request.
