# Supplementary material for: Plasma rich in growth factors in alveolar ridge preservation: randomized, controlled clinical trial
Source: Clin Oral Investig. 2026 Mar 23;30(4):137. doi: 10.1007/s00784-026-06769-z (PMC13009063; doi:10.1007/s00784-026-06769-z)
Supplement: Supplementary file 1 — Supplementary Material 1 [file 784_2026_6769_MOESM1_ESM.docx]

**Plasma Rich in Growth Factors in Alveolar Ridge Preservation: Randomized, Controlled Clinical Trial**

Clinical Oral Investigations

Eduardo Anitua^1,2,3,*^, Alia Murias-Freijo^1,4,5,*^, Joseba Loroño^6^, Markel Loroño^6^, Antonio González-Mosquera^67^, Lucia Anitua^1^, Mohammad H. Alkhraisat^2,3,8^

^1^ Clinica Eduardo Anitua, Vitoria, Spain

^2^ University Institute for Regenerative Medicine & Oral Implantology, UIRMI (UPV/EHU-Eduardo Anitua Foundation), Vitoria, Spain.

^3^ BTI-Biotechnology Institute, Vitoria, Spain.

^4^ Clinica Dental Murias, Bilbao, Spain

^5^ Biomedical Research, Department of Cell Biology and Histology, Medicine and Nursing School, University of the Basque Country UPV/EHU, Leioa, Spain.

^6^ Clinica Dental Loroño, Bilbao, Spain

^7^ Clínica Antonio González Mosquera, A Coruña, Spain

^8^ Oral and Maxillofacial Surgery, Oral Medicine and Periodontics Department, Faculty of Dentistry, University of Jordan, Amman 11942, Jordan.

* Both authors contributed equally

**Corresponding Author** Email: [eduardo@fundacioneduardoanitua.org](mailto:eduardo@fundacioneduardoanitua.org).

Table 1. Baseline buccal wall width measurements obtained at 1, 3, and 5 mm from the alveolar crest. Data were stratified by tooth type and study group

| **Tooth type** | **Treatment** | | **Buccal wall width (mm) measured at** | | |
| --- | --- | --- | --- | --- | --- |
|  |  |  | 1 mm | 3 mm | 5 mm |
| Central incisor | Control | Mean | 0.50 | 0.50 | 0.40 |
|  |  | Median | 0.50 | 0.50 | 0.40 |
|  |  | Std. Deviation | . | . | . |
|  | Test | Mean | 0.75 | 0.70 | 0.95 |
|  |  | Median | 0.75 | 0.70 | 0.95 |
|  |  | Std. Deviation | 0.21 | 0.14 | 0.64 |
| Lateral incisor | Control | Mean | 0.90 | 0.90 | 0.91 |
|  |  | Median | 0.8000 | 0.90 | 10.0000 |
|  |  | Std. Deviation | 0.32 | 0.37417 | 0.48 |
|  | Test | Mean | 0.45 | 0.50 | 0.40 |
|  |  | Median | 0.45 | 0.50 | 0.40 |
|  |  | Std. Deviation | 0.21 | 0.28 | 0.14 |
| Canine | Test | Mean | 0.53 | 0.50 | 0.40 |
|  |  | Median | 0.50 | 0.40 | 0.50 |
|  |  | Std. Deviation | 0.15 | 0.26 | 0.26 |
|  | Total | Mean | 0.53 | 0.50 | 0.40 |
|  |  | Median | 0.50 | 0.40 | 0.5000 |
|  |  | Std. Deviation | 0.15 | 0.26 | 0.26 |
| First premolar | Control | Mean | 1.00 | 0.96 | 0.77 |
|  |  | Median | 1.09 | 0.80 | 0.60 |
|  |  | Std. Deviation | 0.32 | 0.38 | 0.31 |
|  | Test | Mean | 0.77 | 0.95 | 0.82 |
|  |  | Median | 0.76 | 0.80 | 0.92 |
|  |  | Std. Deviation | 0.27 | 0.42 | 0.28 |
| Second premolar | Control | Mean | 1.99 | 2.31 | 1.59 |
|  |  | Median | 0.90 | 10.66 | 0.63 |
|  |  | Std. Deviation | 1.93 | 1.94 | 1.85 |
|  | Test | Mean | 1.11 | 1.60 | 1.44 |
|  |  | Median | 1.05 | 1.40 | 1.12 |
|  |  | Std. Deviation | 0.13 | 0.61 | 0.87 |
